# Impact of the SARS-CoV-2 Pandemic on the Epidemiology of *Streptococcus pyogenes*: A Five-Year Retrospective Study

**DOI:** 10.3390/microorganisms12122403

**Published:** 2024-11-23

**Authors:** Patricia Brañas, Fabiola Fontenla, María Victoria Castaño-Amores, Raúl Recio, Irene Muñoz-Gallego, Jennifer Villa, Esther Viedma, Lola Folgueira

**Affiliations:** 1 Microbiology Department, Hospital Universitario 12 de Octubre, Avda. Córdoba s/n, 28041 Madrid, Spain; 2 Biomedical Research Institute imas12, Hospital Universitario 12 de Octubre, 28041 Madrid, Spain; 3 Department of Medicine, School of Medicine, Universidad Complutense, 28040 Madrid, Spain

**Keywords:** *Streptpcoccus pyogenes*, group A *Streptococcus* (GAS), pandemic, invasive infection, epidemiology

## Abstract

The SARS-CoV-2 pandemic significantly affected the epidemiology of *Streptococcus pyogenes*, a pathogen associated with various clinical presentations such as pharyngitis, scarlet fever, and invasive diseases. This study analyzed the incidence and characteristics of *S. pyogenes* infections between 2018 and 2023, examining 915 cases categorized as either respiratory or non-respiratory. Respiratory infections predominantly affected children, accounting for 76% of cases, with a median age of 5 [3, 8] years, while non-respiratory infections were more common in adults, with a median age of 46.5 [34, 64] years. Invasive respiratory infections, such as pneumonia and empyema, were more frequent in children (54.8%), whereas invasive non-respiratory infections, such as primarily cellulitis, were predominantly seen in adults (90.5%). A sharp decline in *S. pyogenes* infections was observed during the pandemic, with respiratory cases decreasing tenfold in 2020 compared to the previous year, and non-respiratory cases experiencing a twofold reduction. However, infection rates returned to pre-pandemic levels by 2022 and 2023, with a notable resurgence of invasive respiratory infections in children following a public health alert in the United Kingdom in late 2022. These findings highlight distinct infection patterns between pediatric and adult populations and emphasize the significant impact of the pandemic on respiratory infections, particularly in children.

## 1. Introduction

The SARS-CoV-2 pandemic has had a profound impact on healthcare systems worldwide, affecting the incidence and management of various infectious diseases [1,2]. Group A Streptococcus (GAS), or *Streptococcus pyogenes*, is a bacterium responsible for a wide range of infections, from mild conditions such as pharyngitis and scarlet fever to severe invasive diseases such as necrotizing fasciitis and streptococcal toxic shock syndrome [3]. The pandemic may have indirectly affected the epidemiology of *S. pyogenes* infections due to changes in healthcare-seeking behavior, public health interventions, and the reduced circulation of certain respiratory viruses such as influenza and respiratory syncytial virus (RSV) [4,5].

This study aims to assess the incidence of *S. pyogenes* infections before and during the SARS-CoV-2 pandemic, with a particular focus on analyzing differences in infection patterns based on the origin of the infection (respiratory vs. non-respiratory). The goal is to better understand the pandemic’s indirect effects on this significant pathogen. Additionally, it seeks to describe the different clinical presentations between pediatric and adult populations, with particular attention to invasive diseases caused by *S. pyogenes*.

## 2. Material and Methods

This retrospective study was conducted at Hospital Universitario 12 de Octubre, a tertiary care facility in southern Madrid, Spain, serving a population of 460,000 individuals. All patients who presented to the Emergency Department, clinical services, or primary care between 1 January 2018, and 31 December 2023, with *S. pyogenes* infections confirmed by the isolation of the bacterium from clinical samples via culture were included. Only one isolate per patient was considered.

Two infection origins were distinguished: respiratory and non-respiratory (primarily skin and soft tissues). Primary bacteremias with no identifiable focus were categorized as having an unknown origin. Respiratory samples included respiratory secretions, pharyngeal, conjunctival, otic, pleural fluids, and blood cultures. Non-respiratory specimens included skin swabs, biopsies, various bodily fluids, pus/abscesses, corneal swabs, and blood cultures. Invasive GAS infections were defined as illnesses associated with the detection of GAS in a normally sterile site.

The medical records of all patients with invasive disease were reviewed. Demographic (age, gender, and nationality), comorbidity, and mortality (death within 30 days of onset of symptoms) data were collected and analyzed. Underlying conditions included diabetes, hypertension, obesity, immunosuppression, cancer, hematological malignancy, HIV, transplant, chronic lung disease (chronic obstructive pulmonary disease, asthma), heart disease (congestive heart failure or coronary artery disease), chronic kidney disease, chronic liver disease, skin wound or injury, and a recent surgery (prior one month).

The potential correspondence of a probable SARS-CoV-2 infection in the 30 days preceding the onset of clinical symptoms was assessed for all invasive infections. Additionally, in cases of invasive respiratory GAS disease, the presence of a prior viral infection with other respiratory viruses (RSV, influenza A/B, rhinovirus, human metapneumovirus, or parainfluenza virus) was also reviewed.

Patients were categorized into pediatric (<18 years) and adult (≥18 years) groups. To evaluate the impact of the SARS-CoV-2 pandemic on *S. pyogenes* infections, we compared incidence rates before (January 2018, to February 2020) and during the pandemic (March 2020, to December 2023). Furthermore, a review and analysis were conducted of the most significant non-pharmaceutical and public health measures implemented in the community of Madrid to control the spread of COVID-19 throughout the study period. Finally, we examined the rise in pediatric pneumonia cases that coincided with the public health alert in the United Kingdom at the end of 2022.

In order to assess the circulation of SARS-CoV-2 within our region and the incidence of COVID-19 cases from March 2020 to December 2023, data were collected from all patients tested for SARS-CoV-2 via real-time PCR. The results were then analyzed to determine the positivity rate throughout this period.

For statistical analysis, quantitative data were presented as medians with interquartile ranges, while qualitative variables were expressed as absolute and relative frequencies. Categorical variables were compared using the χ^2^ test, while continuous variables were analyzed using either the Student’s *t*-test or the Mann–Whitney U test, as appropriate. A *p*-value < 0.05 was considered statistically significant. Ethical approval was obtained from the Research Ethics Committee of our institution (*CEIm: 24/373*).

## 3. Results

A total of 915 patients were included in the study, with 58% (531/915) being pediatric. Of these, 545 cases were classified as respiratory, 359 as non-respiratory, and 11 as having an unknown origin (primary bacteremias).

### 3.1. Respiratory Source

Patients with respiratory *Streptococcus pyogenes* infections were predominantly pediatrics, representing 76% (414/545) of cases. The median age [interquartile range] of children with respiratory diseases was 5 [3, 8] years, with 52.9% treated in the Emergency Department. Among adults (N = 131), the median age was 34 [26, 49] years, and 55.7% were treated in primary care or outpatient clinics. Respiratory cases occurred slightly more in males, comprising 53.4% of the total.

**Figure 1:**
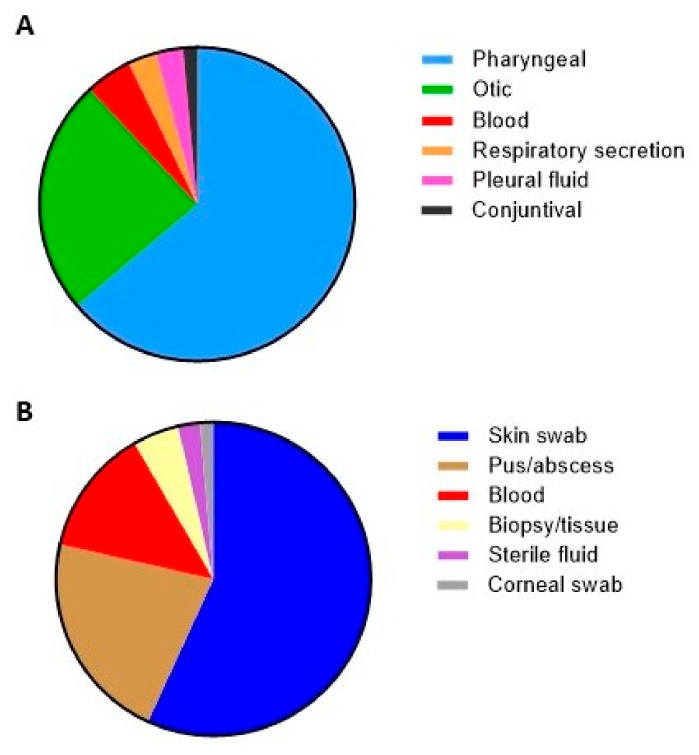
Distribution of *Streptococcus pyogenes* isolates by sample type: (**A**) respiratory source; (**B**) non-respiratory source. **Footnote:** *S. pyogenes* was isolated from 11 blood cultures with an unknown focus. These samples are not included in the graphs.

**Figure 2:**
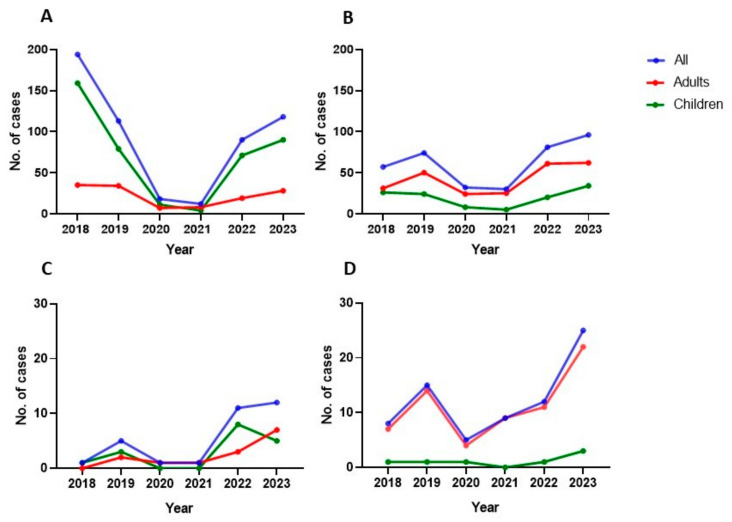
Comparative study of GAS infection in children and adults: (**A**) all cases of respiratory infection; (**B**) all cases of non-respiratory infection; (**C**) invasive respiratory disease; (**D**) invasive non-respiratory disease. **Footnote**: A decrease was noted in 2019 compared to 2018 at the expense of pharyngeal samples from the Pediatric Emergency Department (up to this point, positive antigenic rapid tests for GAS were sent to Microbiology for culture) (panel (**A**), green line).

**Figure 3:**
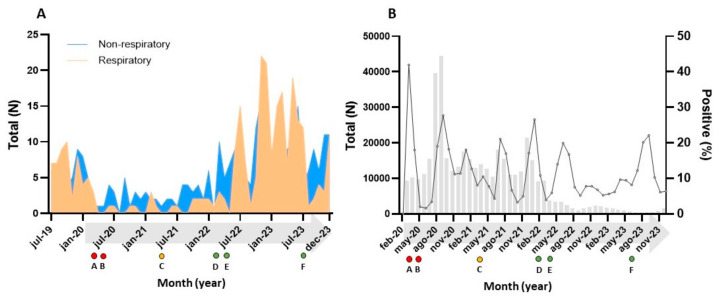
(**A**) Seasonal evolution of *S. pyogenes* throughout the study period based on the infection source. (**B**) Distribution of patients with suspected SARS-CoV-2 infection and positivity rate since the beginning of the pandemic. **Footnote:** Significant events concerning the non-pharmacological and public health measures to contain COVID-19 in the community of Madrid are shown below the arrow for each panel. **A. march-20**: Onset of the COVID-19 pandemic. State of emergency declared. National lockdown (RDL 463/2020, 14 March); **B. may-20:** End of lockdown. Small group gatherings allowed, mobility restrictions, mask use required (O. SND/422/2020, 19 May); **C. march-21:** End of state of emergency. Mandatory mask use in public places (LRJSP 2/2021, 29 March). Vaccination underway since January 2021; **D. february-22:** End of mandatory mask use outdoors (RDL 115/2022, 8 February); **E. april-22:** End of mandatory mask use indoors (RDL 268/2022, 19 April); **F. july-23:** End of mask use requirement in healthcare and social care facilities (O. SND/726/2023, 4 July).

The primary sources of respiratory infections were pharyngeal (N = 348) and otic specimens (N = 132) (Figure 1A). A significant reduction in *S. pyogenes* infections was observed after the onset of the SARS-CoV-2 pandemic, with only 18 cases in 2020 and 12 in 2021, compared to previous years. However, by 2022 and 2023, the number of infections returned to pre-pandemic levels, reaching 118 in 2023 (Figure 2 and Figure 3).

#### Respiratory Invasive Infection

Of the respiratory cases, 5.7% (31/545) were classified as invasive, with most occurring in children (54.8%, 17/31). Pediatric patients with invasive infections had a lower median age of 2 [1, 4] years compared to those with non-invasive respiratory infections, whose median age was 5 [3, 8] years (*p* < 0.001). In adults, invasive cases were associated with a significantly higher median age of 59.5 [48, 69] years, compared to 32 [25, 40] years for non-invasive cases (*p* < 0.001).

**Figure 4:**
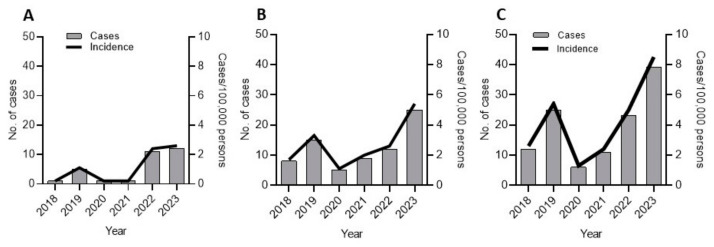
Annual number of cases of invasive GAS and incidence (cases per 100,000 persons) between 2018 and 2023: (**A**) respiratory infection; (**B**) non-respiratory infection; (**C**) all cases.

Overall, 4.1% (17/414) of pediatric respiratory cases were invasive, compared to 10.7% (14/131) in adults (*p* = 0.008). A notable gender difference was observed, with males representing 71% of invasive cases, especially in the adult group (*p* = 0.011). A significant increase in invasive cases was recorded in 2022 and 2023, following a public health alert in the UK, driven mainly by a rise in pediatric pneumonia cases. The incidence rose from 0.2 per 100,000 in 2021 to 2.4 in 2022, reaching 2.6 in 2023 (Figure 4A). Most pediatric invasive cases (13/17) occurred between late 2022 and early 2023, with none recorded in 2020 or 2021. Adult cases also increased, with 7 reported in 2023 (Figure 2C).

Among the invasive cases, 64.5% (20/31) presented as pneumonia, with 17 (85%) of these involving empyema. The remaining 35.5% of invasive cases included five patients with other lower respiratory tract infections, five with complicated cervical abscesses, one with a frontal abscess secondary to complicated sinusitis, and one patient with otomastoiditis.

A preceding viral infection was documented in 48.4% (15/31) of the invasive cases, 13 of whom were children. These included four cases of respiratory syncytial virus, four of influenza A, two of influenza B, one of SARS-CoV-2, and four cases caused by other viruses. Notably, co-detection with two or more viruses was observed in seven pediatric patients, with rhinovirus being the most frequently identified in five cases. No viral pathogen was detected in nine patients, and viral testing was not performed in the remaining seven cases.

In terms of clinical characteristics, only 5.9% of pediatric cases had an underlying condition (one case of hyaline membrane disease), whereas 78.6% (11/14) of adult cases presented with comorbidities, the most frequent being hypertension (N = 7) and chronic lung disease (N = 5). Among pediatric patients, 82.3% required ICU admission, and 5.9% died. In contrast, 28.6% of adult patients were admitted to the ICU, with a mortality rate of 14.3% (Table 1).

### 3.2. Non-Respiratory Source

Non-respiratory *S. pyogenes* infections were more frequent in adults, accounting for 68.5% (246/359) of cases. The median age of adults with non-respiratory infections was 46.5 [34, 64] years, while for pediatric patients it was 5 [3, 8] years. Most cases (234/359) were seen in the Emergency Department, with males representing 60.7% of this group.

The most common sources of non-respiratory infections were skin exudates (56.8%), pus/abscesses (21.7%), and blood cultures (13.1%) (Figure 1B). Similar to respiratory cases, non-respiratory diagnoses showed a moderate decline in 2020 (n = 32) compared to pre-pandemic levels (69 in 2019). This trend continued in 2021 (n = 29), but numbers rose again in 2022 and 2023, exceeding pre-pandemic totals (Figure 2 and Figure 3).

#### Non-Respiratory Invasive Infection

Seventy-four cases (20.6%) of non-respiratory infections were classified as invasive, most of which occurred in adults (90.5%, 67/74). Adults with invasive non-respiratory infections had a significantly older median age (59 [45, 78] years) compared to those with non-invasive infections (42 [31, 59] years, *p* < 0.0001). Males accounted for 56.8% of invasive non-respiratory cases.

Overall, 27.2% (67/246) of adult non-respiratory infections were classified as invasive, compared to 6.2% (7/113) in pediatric patients (*p* < 0.0001). Invasive non-respiratory cases dropped from 15 in 2019 to 5 in 2020, then experienced a slight increase in 2021 (n = 9) before surging to 25 cases in 2023. The incidence in 2023 reached 5.4 per 100,000 inhabitants, the highest recorded during the study period (Figure 4B).

The clinical manifestations of these invasive diseases included cellulitis (n = 42), necrotizing fasciitis (n = 12), surgical wound infections (n = 6), septic arthritis (n = 6), gynecological infections (n = 4), abdominal infections (n = 2), and central nervous system infections (n = 2).

Consistent with findings for respiratory infections, only one pediatric patient (14.3%) with invasive disease of non-respiratory origin had an underlying condition (Tetralogy of Fallot); 71.4% of these patients were admitted to the ICU, with no deaths reported. In contrast, 65.7% (44/67) of adult patients had at least one comorbidity, most commonly hypertension (n = 30) and diabetes (n = 25). Additionally, 52.2% (35/67) presented with a skin wound or injury that likely served as the entry point for *S. pyogenes* infection. The mortality rate among the adult population was 13.4% (Table 1).

Finally, regarding SARS-CoV-2 among non-respiratory invasive cases, only one positive result was obtained in a single patient (who died due to septic shock caused by abdominal *S. pyogenes* infection); 27 patients tested negative, and testing was not performed for the remaining 22 patients since the beginning of the pandemic.

## 4. Discussion 

Our study provides valuable insights into the epidemiology of *S. pyogenes* in our region over the past five years, illustrating notable shifts in its dynamics following the emergence of SARS-CoV-2. Previous research has documented significant changes in the circulation of respiratory-transmitted microorganisms during the COVID-19 pandemic [4,6]. A recent regional study [7] highlighted the disappearance of influenza viruses during the 2020 and 2021 seasons and the seasonal displacement of RSV to the summer of 2021. In parallel, we observed a marked decline in *S. pyogenes* infections during 2020 and 2021, coinciding with high levels of SARS-CoV-2 circulation and rigorous public health interventions aimed at curbing its spread (Figure 3). While reductions in *S. pyogenes* circulation during the pandemic have been reported elsewhere [8,9], these studies did not specifically analyze the impact by infection site.

The nasopharyngeal mucosa and skin are the primary sites of asymptomatic GAS colonization [3,10]. Our data reveal a striking 10-fold reduction in respiratory infections in 2020 compared to the previous year, along with a more modest 2-fold reduction in non-respiratory cases. This suggests that the non-pharmaceutical interventions implemented to control COVID-19 may have significantly curtailed respiratory transmission of *S. pyogenes*.

Microbial interactions were also influenced by the pandemic. Co-detection of certain bacterial agents alongside respiratory viruses is a recognized risk factor for more severe disease, as previously reported [5,11]. Different studies have assessed the relationship between the circulation of various respiratory viruses and the development of invasive pneumococcal disease during the pandemic. These studies report similar levels of nasopharyngeal pneumococcal carriage in both pre-pandemic and pandemic periods, suggesting that the decline in invasive disease cases during COVID-19 may be more closely associated with the reduction in viral infections than with the containment measures implemented by health authorities [5,12]. Further studies are needed to better understand the impact of viral agents during the pandemic on the pharyngeal *S. pyogenes* carriage state.

The reduced circulation of respiratory viruses in our region [7] likely contributed to the drop in invasive GAS respiratory infections in 2020 and 2021. However, coinciding with the resurgence of seasonal patterns of major respiratory viruses, a significant increase in pediatric cases of pneumonia and empyema was observed in late 2022 and 2023, in alignment with the public health alert issued in the UK [9,13]. Of these cases, 76.9% were associated with a concurrent viral infection alongside the onset of invasive GAS disease.

The spread, virulence, and outbreaks of *S. pyogenes* are also driven by circulating bacterial clones. While we did not characterize specific strains in this study, previous research has highlighted the wide variety of *emm* types, with type 1 being the most prevalent across multiple studies [14,15,16]. In particular, the hypertoxigenic *emm1* lineage-M1UK has surged in Europe in recent years, often linked to more severe disease [17,18].

Our findings indicate that GAS respiratory infections were predominantly observed in pediatric patients, whereas non-respiratory infections were more common among adults. Notably, the majority of children with an invasive disease were otherwise healthy, with no underlying conditions—a pattern that has been documented in prior studies [19,20,21]. Additionally, most pediatric patients required ICU admission, although mortality rates remained low. In contrast, adult patients with invasive diseases largely presented with pre-existing conditions, as previously reported [15,21,22]. Of particular note, non-respiratory infections in adults were frequently associated with a skin wound or injury serving as the primary entry point for *S. pyogenes*, a finding consistent with earlier studies [23].

This study has some limitations. First, as a retrospective study, data collection may be inconsistent, potentially leading to under-reporting or missed identification of invasive disease cases. Second, for non-invasive disease cases, not all clinical data from the patients were reviewed, raising the possibility that some cases may represent colonization rather than true infections. Third, the number of samples submitted decreased during the early stages of the pandemic, potentially impacting case detection. Finally, we did not include the characterization of circulating clones in our analysis.

However, this study demonstrates significant shifts in the incidence of *S. pyogenes* infections over the years, highlighting a pronounced decline during the pandemic, particularly in respiratory cases. Furthermore, it underscores the distinct epidemiological dynamics of GAS across age groups. Continuous monitoring is essential for ensuring effective preparedness against potential future outbreaks.

## Figures and Tables

**Table 1:** Clinical and demographic characteristics of patients with group A *Streptococcus* infections by source of infection and age group.

Characteristic	Respiratory Source, N (%)		Non-Respiratory Source, N (%)	
	Children (N = 17)	Adults (N = 14)	Children (N = 7)	Adults (N = 67)
Age (years) (median, IQR)	2 [1, 4]	59.5 [48, 69]	8 [7, 9]	59 [44, 75]
Male sex	11 (64.7)	11 (78.6)	7 (100)	35 (52.2)
Nationality				
Spain	15 (88.2)	10 (71.4)	6 (85.7)	50 (74.6)
Latin America	2 (11.8)	4 (28.6)	1 (14.3)	13 (19.4)
Eastern Europe	0	0	0	4 (6)
Underlying conditions				
Diabetes mellitus	0	0	0	17 (25.4)
Heart disease	0	3 (21.4)	1 (14.3)	9 (13.4)
Hypertension	0	7 (50)	0	30 (44.8)
Dyslipidemia	0	2 (14.3)	0	21 (31.3)
Obesity	0	3 (21.4)	0	10 (14.9)
Chronic lung disease	1 (5.9)	5 (35.7)	0	9 (13.4)
Chronic kidney disease	0	0	0	5 (7.5)
Chronic liver disease	0	0	0	2 (3)
Cancer	0	3 (21.4)	0	7 (10.4)
Haematological malignancy	0	0	0	1 (1.5)
Transplant	0	1 (7.1)	0	1 (1.5)
HIV	0	0	0	3 (4.5)
Immunossuppresion	0	2 (14.3)	0	2 (3)
Any underlying condition	1 (5.9)	11 (78.6)	1 (14.3)	44 (65.7)
Recent surgery	0	0	1 (14.3)	5 (7.5)
Skin wound or injury	0	0	1 (14.3)	35 (52.2)
ICU hospitalisation	14 (82.3)	4 (28.6)	5 (71.4)	19 (28.4)
Mortality	1 (5.9)	2 (14.3)	0	9 (13.4)

## Data Availability

The data that support the findings of this study are available on request from the corresponding author. The data are not publicly available because of privacy or ethical restrictions.
